# The complete mitochondrial genome of *Athalia proxima* (Hymenoptera: Tenthredinidae) and phylogenetic analysis

**DOI:** 10.1080/23802359.2019.1687042

**Published:** 2019-11-08

**Authors:** Huimin He, Gengyun Niu, Bin Zhang, Meicai Wei

**Affiliations:** a College of Life Sciences, Jiangxi Normal University, Nanchang, China;; b Key Laboratory for Cultivation and Protection of Non-Wood Forest Trees, Central South University of Forest and Technology, Changsha, China

**Keywords:** Mitogenome, next-generation sequencing, phylogeny, Tenthredinidae, Athalia

## Abstract

The nearly complete mitochondrial genome of *Athalia proxima* is 15852 bp in size, containing 13 protein-coding genes, 22 transfer RNAs, 2 ribosomal RNAs, and a partial control region. Compared to ancestral organization, seven tRNA genes were rearranged. Bayesian and maximum-likelihood trees recovered the monophyly of Tenthredinidae and *A. proxima* was the basal lineage. The mitogenome sequence provided fundamental data for resolving phylogenetic and genetic problems of Tenthredinidae.

*Athalia proxima* (Klug) is a common pest of Chinese cabbage of Brassicaceae with a wide distribution in East Asia. About 102 species and sub-species have been reported in *Athalia* (Taeger et al. 2010; Koch et al. 2015). The systematic position of *Athalia* within Tenthredinoidea is uncertain (Wei and Nie 1998; Schulmeister et al. 2002; Taeger et al. 2010). To obtain basic genetic information regarding *A. proxima* and enrich the Tenthredinoidea species genome resource to promote the phylogeny of Tenthredinoidea, we herein describe the near-complete mitogenome of *A. proxima*.

Specimen (CSCS-Hym-MC0021) available at the Asia Sawfly Museum, Nanchang (ASMN) repository was collected from Mt. Dapan, Pan’an, Zhejiang (28.85°N 120.46°E) in 2015. Whole genomic DNA was extracted from the specimen’s thorax muscle using the DNeasyR Blood & Tissue Kits (Qiagen, Valencia, CA, USA). Genomic DNA was prepared in 150 bp paired-end libraries, tagged and subjected to the high-throughput Illumina Hiseq 4000 platform, yielding a total of 8,985,824 raw reads (SRP223539). DNA sequences were assembled using MitoZ (Meng et al. 2019), and Geneious Prime 2019.2.1 (https://www.geneious.com) using three relative species as reference with the mean depth of coverage across the sequences being 1718, 1981 and 2092, respectively. Annotations were generated in MITOS web server (Bernt et al. 2013) and revised in Geneious Prime when necessary.

A nearly complete mitogenome of 15852 bp was obtained and deposited in GenBank with accession number MN527306. The *trnP* and *trnT* had altered positions, which were detected in *Megalodontes*. The ancestral pattern of *trnI* (+)-*trnQ* (−)-*trnM* (+) clusters were rearranged to *trnC* (+)-*trnW* (+)-*trnQ* (−)-*trnM* (+)-*trnI* (+), which is novel to the basal hymenopterans.

The base composition was 43.7% (A), 11.1% (C), 7.3% (G) and 37.9% (T), with a high A + T content (80.4%) of PCGs, indicating significant A + T bias. The length of the PCGs accounted for 70.7% of the mitogenome *cox1* originated from TTG. Five PCGs originated from ATG whilst another four originated from ATT. The remainder originated from ATA.

The phylogenetic status of *A. proxima* was inferred using BA and ML topologies, performed for protein-coding. The phylograms produced identical topologies (Figure 1). *Athalia proxima* formed a sister clade with Tenthredinidae + Cimbicidae as one of the basal lineages of Tenthredinoidea.

**Figure 1.**
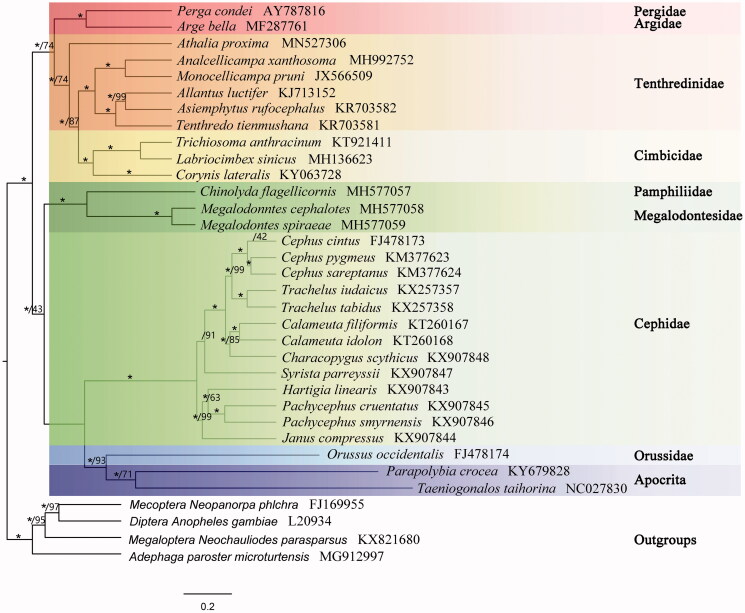
A majority-rule consensus tree inferred Bayesian inference using MrBayes v.3.2.2 (Ronquist et al. 2012) under the GTR + GAMMA model, based on the concentrated PCGs + RNA of 28 individuals of Symphytans, two Apocritans and four outgroups. GenBank accession numbers were provided with species names. DNA sequences (Total 11,393 bp) were aligned in TranslatorX (Abascal et al. 2010). Node numbers show Bayesian posterior probabilities (*=100) and bootstrap. Branch lengths represent the means of the posterior distributions.
